# Simple non-invasive biomarkers of advanced fibrosis in the evaluation of non-alcoholic fatty liver disease

**DOI:** 10.1093/gastro/gou034

**Published:** 2014-07-07

**Authors:** Elliot B. Tapper, Katherine Krajewski, Michelle Lai, Tracy Challies, Robert Kane, Nezam Afdhal, Daryl Lau

**Affiliations:** ^1^Division of Gastroenterology, Beth Israel Deaconess Medical Center, Boston, MA USA; ^2^Division of Radiology, Beth Israel Deaconess Medical Center, Boston, MA, USA; ^3^Division of Pathology, Beth Israel Deaconess Medical Center, Boston, MA, USA

**Keywords:** cirrhosis, non-alcoholic fatty liver disease, non-alcoholic steatohepatitis, aspartate aminotransferase-to-platelet ratio index

## Abstract

**Background and aims.** Non-alcoholic fatty liver disease (NAFLD) is a common, morbid disease with profound implications for the overall health of the patient. We set out to determine the clinical predictors of advanced histology in the referral population.

**Methods.** We performed a retrospective review of all biopsy-proven NAFLD patients, including 358 unique patients first seen between 1996 and 2009. Liver histology and ultrasound images were reviewed prospectively by clinicians who were blinded to clinical information and test indication.

**Results.** Compared with men, women tended to present at an older age (51.4 ± 10.6 *vs* 45.3 ± 11.2 years, *P < *0.001), were more likely to be Caucasian (*P = *0.003), less likely to present with an elevated alanine aminotransferase (ALT) (75.2% *vs* 88.8%), and more likely to have advanced non-alcoholic steatohepatitis (NASH) (44.7% *vs* 29.9%; *P = *0.04) and advanced fibrosis (23.3% *vs* 14.1%; *P = *0.03). In multivariate logistic regression, body mass index (BMI) ≥30 kg/m^2^ (odds ratio (OR) 2.21; 95% confidential interval (CI): 1.23–4.08), female gender (OR 1.76; 95% CI: 1.01–3.10) and aspartate aminotransferase (AST) >40 IU/L (OR 2.00; 95% CI: 1.14–3.55) were associated with a NAFLD activity score >4. The sensitivity and specificity of an AST to platelet ratio index (APRI) >1 for significant fibrosis was 30.0% (95% CI: 17.2–45.4%) and 92.8% (95% CI: 88.2-95.8%), respectively; the likelihood ratio is 4.2. In multivariate logistic regression, APRI >1 was the most significant predictor of advanced fibrosis (OR 3.85; 95% CI: 1.55–9.59). In patients without ultrasound-detected steatosis, 20% had advanced fibrosis and 16.7% had active NASH.

**Conclusion.** Patients with suspected NAFLD should routinely be evaluated for advanced liver disease, including non-invasive indices of fibrosis such as APRI, and serious consideration given to liver biopsy.

## INTRODUCTION

Non-alcoholic fatty liver disease (NAFLD) is a common, morbid disease closely linked to insulin resistance and metabolic syndrome, conditions endemic to the developed world [1]. NAFLD is, in reality, a spectrum. It begins as simple hepatic fat accumulation (steatosis) and by varying mechanisms and insults, a necro-inflammatory state known as non-alcoholic steatohepatitis (NASH) often arises which, in turn, may progress to fibrosis and, later, cirrhosis and hepatocellular carcinoma [2]. As many as 46% of people in the United States of America population have NAFLD, including 74% of diabetics; 12.2% of Americans have NASH [3].

NAFLD is a significant diagnosis for those afflicted. Patients with NAFLD frequently develop advanced liver disease—it is now the third most common indication for liver transplantation [3]. NAFLD is also increasingly recognized as, at least, a marker for cardiovascular disease [4]. Patients with NAFLD—even children—are more likely to die prematurely than matched controls [5, 6]. Accordingly, our patients would be well served by an accurate, reliable, simple and non-invasive method of diagnosis. There is ample evidence to support ultrasound in this role; in a recent meta-analysis, ultrasound was found to have a sensitivity and specificity for steatosis of 84.8% and 93.6%, respectively [7].

Herein we present a study of the entire cohort of biopsy-proven NAFLD/NASH patients seen at our center. We had two aims with this study: first, we sought to describe the clinical predictors of advanced histology in a tertiary referral center population; second, we sought to describe the rate of advanced histology in patients ‘normal’ ultrasound findings.

## PATIENTS AND METHODS

After approval by the Institutional Review Board of the Beth Israel Deaconess Medical Center (BIDMC), all study procedures conformed with the principles outlined in the Declaration of Helsinki. All patients presenting for their first evaluation for unknown liver disease at the Liver Center of BIDMC between 1996 and 2009, with an ultimate clinicopathologic diagnosis of non-alcoholic fatty liver disease after a liver biopsy, were included in the study. All patients were referred to the Liver Center for diagnosis and evaluation of possible liver disease. Patients with viral hepatitis, alcoholic liver disease, hemochromatosis, Wilson disease, drug-induced liver injury and auto-immune hepatitis were excluded. Patient data were abstracted from electronic medical record notations made within two months (before or after) the liver biopsy. These included age, sex, race, body mass index (BMI), aminotransferase levels, platelet counts, and the presence of diabetes and dyslipidemia. An aspartate aminotransferase-to-platelet ratio index (APRI) was calculated by dividing the aminotransferase level by the platelet count. All patients included underwent percutaneous liver biopsy and abdominal ultrasound with the six months prior to the liver biopsy. The database were then maintained in a password-protected Microsoft Excel file.

All patients received a liver biopsy; however only 261 were available for blinded re-review. The liver biopsies were procured in the usual fashion, fixed in formalin and paraffin-embedded at the time of procurement. All biopsies were recalled from archives based on the clinical diagnosis of non-alcoholic fatty liver disease and examined with one hematoxylin and eosin section and a Masson trichrome-stained section. Each biopsy was scored using standardized criteria determined a specialized hepatopathologist, who was blinded to all clinical information as well as the test indication. Disease activity was scored according to a modification of the scheme proposed by Brunt *et al.* [8]. Specifically, NAS activity was scored as the sum of steatosis-, lobular mixed cell inflammation- and balloon degeneration scores, as follows. (i) steatosis: <5%: score 0; 5–33%: score 1; >33–66%: score 2; >66%: score 3; (ii) lobular mixed cell inflammation: no foci: score 0; <2 foci per 200x field, score 1; 2–4 foci per 200x field, score 2; >4 foci per 200 x field, score 3. Balloon degeneration: none: score 0; rare cells: score 1; many cells: score 2. Fibrosis stage was scored as follows: none = Stage 0; perisinusoidal or portal/periportal = Stage 1; perisinusoidal and portal/periportal = Stage 2; bridging fibrosis = Stage 3; cirrhosis = Stage 4 [8]. Active NASH was defined as a NAS score >4 [9].

The ultrasound results were reviewed a second time by two experienced hepatobiliary radiologists, both blinded to the histology, and scored by consensus reads. Normal echogenicity implied a lack of steatosis. Steatosis was detected by increases in liver echogenicity, focal or diffuse, with or without blurring of vascular margins and increased liver-to-right kidney ratio.

Data were collected and stored in a password-protected Microsoft Excel database. Statistical analysis was performed using JMP 8.0.1 software (2009 SAS Institute). Statistical methods were employed according to the nature of the variables involved and included Fisher's exact test, univariate and multivariate logistic regression.

## RESULTS

The complete demographics of—and basic descriptive statistics for—our cohort are detailed in Table 1. We found 358 unique patients seen at our Liver Center between 1996 and 2009, who satisfied the inclusion criteria. Given that it has been shown to affect the presentation of NASH, we divided the population by gender [9]; women presented at an older age, were less likely to have an alanine aminotransferase (ALT) greater than 40 IU/L, more likely to have an NAS score indicative of active NASH (>4) and a biopsy consistent with advanced fibrosis.

**Table 1.** Demographics and clinical characteristics

	Males (*n = *225)	Females (*n = *133)	*P-*value
Age (years)	45.3 ± 11.2	51.4 ± 10.6	**<0.001**
Caucasians, *n* (%)	149 (66.2)	108 (81.2)	**0.003**
BMI (kg/m^2^)	31.7 ± 5.8	33.2 ± 7.1	NS
ALT > 40 IU/L, *n* (%)	200 (88.8)	100 (75.2)	0.03
APRI Score > 1, *n* (%)	22 (9.8)	19 (14.4)	NS
Steatosis by ultrasound, *n* (%)	165 (87.3)	88 (83.8)	NS
Steatosis by histology, *n* (%)	156 (94.0)	87 (91.6)	NS
Lobular inflammation, *n* (%)	145 (88.4)	80 (85.1)	NS
Ballooning degeneration, *n* (%)	112 (68.3)	73 (77.7)	NS
NAS ≥5, *n* (%)	49 (29.9)	42 (44.7)	0.04
Hepatic fibrosis, *n* (%)	108 (66.3)	63 (70.0)	NS
Stage 3 or 4 fibrosis, *n* (%)	23 (14.1)	21 (23.3)	**0.03**

The bolded values listed are significant results. All the percentages listed for ultrasound and histological parameters reflect the proportion of patients with primary data available for blinded secondary review. ALT = alanine aminotransferase, APRI = aspartate aminotransferase (AST)-to-platelet ratio index, BMI = body mass index, NAS = non-alcoholic fatty liver disease (NAFLD) activity score, NS = not significant.

We first sought to characterize the ability of standard assessments to predict advanced histology. Given that the predictors of NASH and advanced fibrosis may be different, the latter being a *sequela* of the former, we addressed these separately. In Table 2, we detail the univariate and multivariate associations with a NAS score greater than 4 (active NASH) as well as a Meta-analysis of Histological Data in Viral Hepatitis (METAVIR) stage greater than 2 (advanced fibrosis). In this referral population, it turns out that a BMI consistent with obesity and an AST >40 IU/L were associated with NAS >4, whilst age and an APRI >1 were associated with advanced fibrosis. Further, the sensitivity and specificity of an APRI greater than 1 for significant fibrosis were, respectively, 30.0% (95% CI: 17.2–45.4%) and 92.8% (95% CI: 88.2–95.8%). The likelihood ratio was 4.2.

**Table 2.** Clinical predictors of advanced NASH and advanced fibrosis

Variables	Predictors of advanced NASH (NAS >4)		Predictors of advanced fibrosis (METAVIR >2)	
	Odds ratio (95% CI)		Odds ratio (95% CI)	
	Univariate	Multivariate	Univariate	Multivariate
Age >median (47 years)	0.98 (0.59–1.63)	–	**2.30 (1.18–4.63)**	**2.01 (1.00–4.15)**
Gender (female)	**1.86 (1.10–3.15)**	**1.76 (1.01–3.10)**	1.82 (0.94–3.53)	–
BMI (obese; ≥30 kg/m^2^)	**2.44 (1.38–4.46)**	**2.21 (1.23–4.08)**	1.14 (0.57–2.34)	–
ALT (>40 IU/L)	0.95 (0.45–1.94)	–	0.95 (0.41–2.49)	–
AST (>40 IU/L)	**2.03 (1.21–3.46)**	**2.00 (1.14–3.55)**	**2.72 (1.36–5.76)**	1.79 (0.83–4.01)
ALT/AST < 1	1.72 (0.79–3.73)	–	2.31 (0.94–5.35)	–
APRI >1	1.70 (0.76–3.76)	–	**5.45 (2.35–12.61)**	**3.85 (1.55–9.59)**

ALT = alanine aminotransferase, APRI = AST-to-platelet ratio index, AST = aspartate aminotransferase, BMI = body mass index, NAS = non-alcoholic fatty liver disease (NAFLD) activity score.

**Figure 1.**
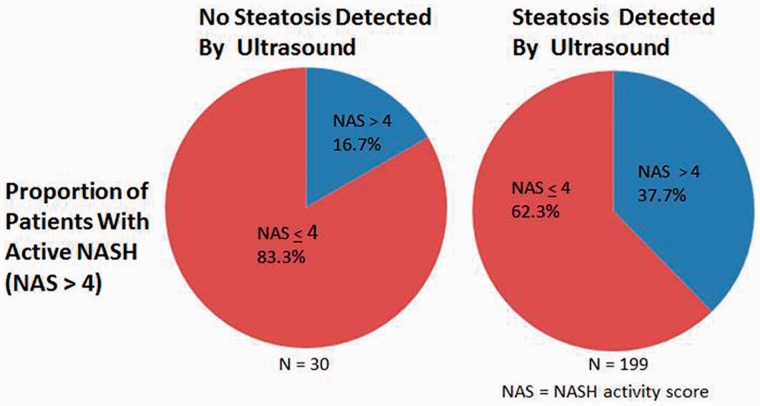
Active non-alcoholic hepatitis (NASH) in patients with and without ultrasound-detected steatosis.

We next turned to our analysis of histological associations with steatosis detected by ultrasound. A total of 229 patients had both their original ultrasound and liver biopsy available for secondary review. While ultrasound reads often suggested that advanced liver disease could not be excluded, overt radiological signs of cirrhosis were not detected in this population. When the ultrasound detected steatosis, this finding was 93.8% sensitive (95% CI: 85.4–97.7%) for an NAS score >4, with a positive predictive value (PPV) of 37.7% (95% CI: 31.0–44.8%). Overall, 199 patients had steatosis observed on ultrasound, of whom 75 (37.7%) had biopsy results consistent with active NASH (NAS >4). Conversely, 30 patients with steatosis on biopsy did not have steatosis detected by ultrasound, 5 (16.7%) of whom had active NASH (NAS > 4). Viewed a different way, the proportion of patients with active NASH with and without ultrasound-detected steatosis is represented in Figure 1.

**Figure 2.**
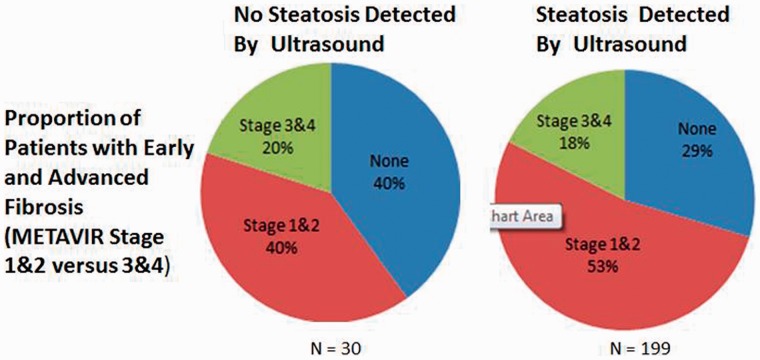
Advanced fibrosis in patients with and without ultrasound-detected steatosis.

A similar result was obtained when examining the association of ultrasound-detected steatosis and advanced fibrosis (Figure 2). The finding of steatosis on ultrasound predicted advanced fibrosis on biopsy with a sensitivity of 85.4% (95% CI: 70.1–93.9%) with a PPV of 17.8% (95% CI:12.8–23.9%). Thirty-five of 199 patients with ultrasound-detected steatosis (17.6%) had advanced fibrosis, while 6 of 30 patients without ultrasound-detected steatosis (20%) had advanced fibrosis (Figure 2). The difference in the METAVIR stage between patients with and without ultrasound-detected steatosis was not significant.

## DISCUSSION

In this study of patients with NAFLD/NASH, evaluated at a tertiary referral center, we present two core findings.

Firstly, liver enzymes, a platelet count and an ultrasound—the basic, non-invasive evaluation of suspected fatty liver disease—can effectively predict the histological changes concordant with NASH and its *sequelae*. At least in the context of the referral population, female sex, BMI >30 kg/m^2^ and elevated AST are associated with active NASH. Meanwhile, age and the APRI score, derivative of the AST and platelet count, is a useful metric for the non-invasive determination of advanced fibrosis in the referral NASH population. The likelihood ratio that we present for an APRI score greater than 1 (14.8) suggests that it may be a potent clinical tool. Others have examined the APRI score with mixed results [11–14]. We found that the sensitivity and specificity of an APRI greater than 1 was 30.0% (95% CI: 17.2–45.4%) and 92.8% (95% CI: 88.2–95.8%). These values are consistent with the 27% and 89% derived from the 145-patient cohort studied by the group from Newcastle-upon-Tyne [13]. The import of this test is significant. Longitudinal data from a large epidemiological study performed in the United States of America showed that the APRI is independently associated with mortality [14]. In our study, our results confirm the utility and general applicability of this score in the American referral population. Risk stratification for advanced histology can be performed using readily available clinical information.

Secondly, while the ultrasound scan may be a critical component in the evaluation of patients referred for possible NAFLD/NASH, it often does not facilitate the identification of advanced liver disease. Indeed, 17.6% of patients with ultrasound-diagnosed steatosis—roughly one of every six—had advanced fibrosis, as did one of every five patients without steatosis detected on ultrasound. Similarly, 16.7%, or one of every six patients without ultrasound-detected steatosis had an NAS score >4. The key importance of this finding is that the ultrasound scan cannot be the last step in evaluation. Presently, only a biopsy can diagnose NASH and it is instructive that one patient in six had a high NAS score without ultrasound-detected steatosis. Accordingly, these data make the lack of steatosis on ultrasound scan less reassuring, especially for patient risk factors for advanced histology (e.g. an obese female with an elevated AST). The role of biopsy in these patients is clear and supported by society (American Association for the Study of Liver Disease) guidelines [1]. Conversely, unlike for the determination of NASH, there are readily available biomarkers to determine the presence of advanced fibrosis. Our data support commencement of this evaluation with an APRI score, regardless of ultrasound findings.

The limitations of our study are based largely on its retrospective design. Given that we utilized clinical data, and given the lack of routine albumin testing, we could not use certain metrics such as the NAFLD fibrosis score [15]. Additionally, our selection criteria required, and were therefore biased by, (i) referral to the liver center and (ii) a liver biopsy. It is important to remember that these results are specifically intended to describe features of the referral population. We addressed the variability and biases due to clinical practice by prospectively reviewing the ultrasounds and histology using radiologists and a pathologist who were blinded to the clinical data. Unfortunately, although we used blinded repeat review to aid in the general applicability of our findings, some of the clinical specimens were not available and thus contemporary interpretation of biopsies or ultrasounds could not be carried out for all patients.

In summary, NAFLD/NASH is a common, morbid disease and, as such, is a significant diagnosis for our patients. In support of available guidelines, when evaluating patients with possible NAFLD/NASH, one must pay attention to the risk of advanced fibrosis. Given that basic blood tests and ultrasound scans are routinely obtained when patients present for evaluation of possible NAFLD, this information can be easily applied to risk stratification.
